# The Role of Social Connection/engagement Predicts Changes in Depressive Symptoms and IADL in Stroke

**DOI:** 10.1093/geroni/igab046.3320

**Published:** 2021-12-17

**Authors:** Joanne Elayoubi, Monica Nelson

**Affiliations:** 1 USF, land o lakes, Florida, United States; 2 University of South Florida, Brandon, Florida, United States

## Abstract

Social connections/engagement have been found to be potentially protective against depression and declines in physical functioning. We examined whether social connection/engagement was protective against depression and functional decline after stroke. Participants were 898 individuals with incident stroke from the Health and Retirement Study between 1998-2012. Multilevel modeling was used to examine how social connection/engagement were associated with trajectories of depressive symptoms and limitations with instrumental activities of daily living (IADLs). Models controlled for age, gender, education, and race. In addition, analyses with depressive symptoms as outcome controlled for functional limitations with ADLs. Participants who were lonely and did not have friends in their neighborhood pre-stroke had more depressive symptoms at the time of stroke. Participants with close children pre-stroke showed less increase in depressive symptoms over time. Within-person increase in loneliness and within-person decline in providing help were related to more depressive symptoms post-stroke. Participants who felt lonely and did not provide help pre-stroke had more IADL limitations at the time of stroke. Smaller pre-stroke household size and pre-stroke volunteering were associated with less increase in IADL limitations with stroke. Within-person increase in having friends and providing help after stroke were associated with fewer IADL limitations post-stroke. Taken together, these findings suggest that social connection/engagement may buffer the negative psychological and physical outcomes of a stressful event such as stroke.

